# Ionic Liquid-Assisted Grinding: An Electrophilic Fluorination Benchmark

**DOI:** 10.3390/molecules26195756

**Published:** 2021-09-23

**Authors:** Pavel A. Zaikin, Ok Ton Dyan, Innokenty R. Elanov, Gennady I. Borodkin

**Affiliations:** 1 Vorozhtsov Novosibirsk Institute of Organic Chemistry, Siberian Branch of Russian Academy of Sciences, 9 Acad. Lavrentiev Ave., 630090 Novosibirsk, Russia; elanov@nioch.nsc.ru (I.R.E.); gibor@nioch.nsc.ru (G.I.B.); 2 Department of Natural Sciences, Novosibirsk State University, 2 Pirogov St., 630090 Novosibirsk, Russia

**Keywords:** aromatic substitution, electrophilic substitution, fluorine, ionic liquids, solvent-free synthesis, mechanochemistry

## Abstract

We demonstrated the influence of liquid additives on the rate and selectivity of mechanochemical fluorination of aromatic and 1,3-dicarbonyl compounds with F-TEDA-BF_4_. Substoichiometric catalytic quantities of ionic liquids speed up the reaction. We proposed an improved protocol for ionic liquids-assisted fluorination that allows easy and efficient isolation of fluorinated products by vacuum sublimation. A careful choice of additive results in high yields of fluorinated products and low E-factor for the overall process. Here, we report a benchmarking study of various ionic liquids in comparison with representative molecular solvents. A lower viscosity of ionic liquid additive is typically associated with higher yields and a higher degree of difluorination. Ionic liquids with fluorous anions (triflate and triflimide) are shown to be the most efficient catalysts for ionic liquid-assisted grinding.

## 1. Introduction

**Scheme 1:**
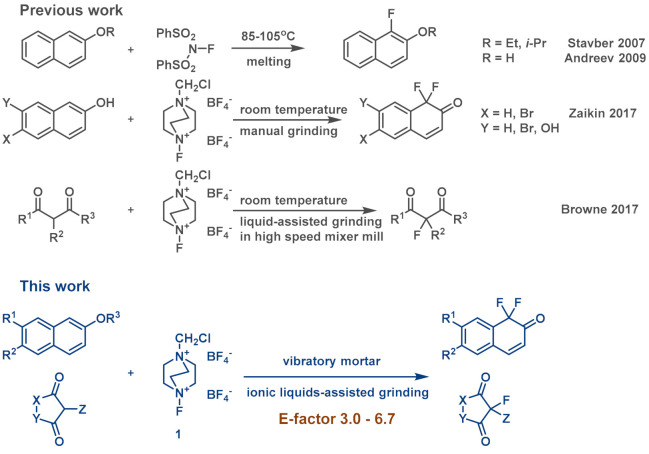
Solvent-free electrophilic fluorination of aromatic and 1,3-dicarbonyl compounds.

Organofluorine compounds play an important role in the development of new materials, modern drugs, and efficient agrochemicals. Many modern fluorine-containing drugs contain fluoroaromatic moieties [1]. Therefore, the development of methods of incorporating C–F bonds in organic molecules attracts much attention [2]. One of the prospective methods of the synthesis of fluoroaromatic compounds is direct electrophilic fluorination of C–H bonds with NF-reagents [3,4]. Such reactions are typically carried out in polar organic solvents like acetonitrile or DMF, and the development of greener alternatives is on the agenda [5]. Many attempts have been made to develop efficient and green fluorination reactions, including reactions in aqueous media [6,7,8], ionic liquids [9,10], and finally, under solvent-free reaction conditions [11,12,13]. We have recently published a solvent-free protocol for the fluorination of phenols and naphthols by manual grinding with mortar and pestle (Scheme 1) [14]. In addition to being tedious, manual grinding is also less reproducible in comparison with mechanical grinding. Several approaches have been proposed for mechanochemical fluorination, but the use of high-speed mixer mills greatly limits the scale-up capabilities of the method [15,16]. The use of continuous reactive extrusion apparently removes the scale-up restrictions but requires huge amounts of grinding additives to be performed [17]. In the present study, we first aimed to translate the mechanochemical fluorination from manual grinding to a mechanical setup that resembles classical grinding with mortar and pestle. Our second goal was the choice of a proper grinding additive to speed up fluorination. As shown in the literature, the use of grinding additives can strongly affect the reaction rates and the selectivity of fluorination [18]. In our previous paper, we used solvent-free fluorination in conjunction with vacuum sublimation of the products directly from the reaction mixture; the process did not involve any solvent at any stage [14]. Therefore, we aimed to use low-volatile additives, and we considered ionic liquids as promising grinding additives. Ionic liquids currently attract much attention due to their polarity, dissolution properties, thermal stability, negligible vapor pressure for the use as reaction media, extraction, or chromatographic separation [19,20]. Recently, ionic liquids were also used as promoters of electrophilic fluorination in organic solvents [21].

Here, we report a first comparison of various ionic liquids as grinding additives in mechanochemical electrophilic fluorination of aromatic and 1,3-dicarbonyl compounds with F-TEDA-BF_4_ **1** in a vibratory mortar grinder. A combination of mechanochemical ionic liquid-assisted grinding with a vacuum sublimation of the product provided low E-factor for the studied reaction. An E-factor of 3 was the lowest achieved. The fluorination reaction was scaled up to 20 mmol and was adapted for the fluorination of the anti-inflammatory drug naproxene.

## 2. Results

**Scheme 2:**
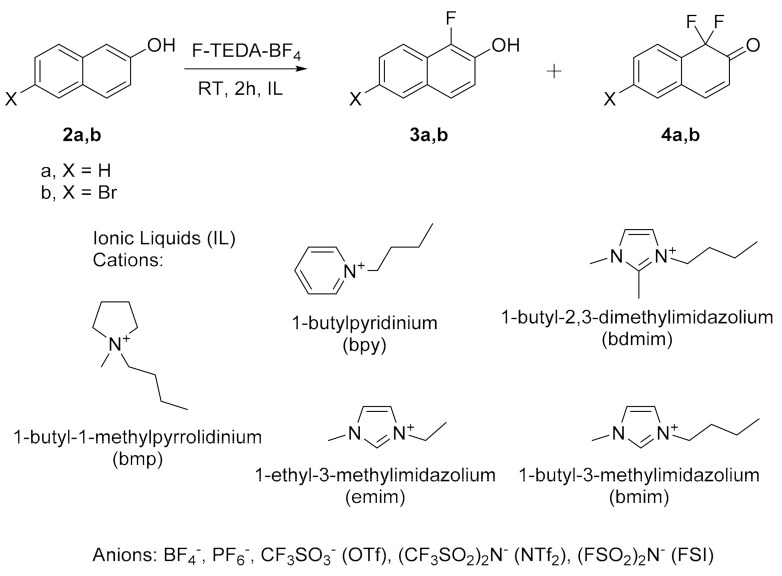
Mechanochemical electrophilic fluorination of naphthols in the presence of grinding additives.

In the first step, we adapted a solvent-free fluorination by manual grinding to a mechanical vibratory mortar grinder. We chose 2-naphthol **2a** as a model substrate that proved to have high activity in electrophilic fluorination and particularly in mechanochemical fluorination [14]. The choice of substrate was also associated with the increased importance of fluorinated naphthalene derivatives in the synthesis of nematic liquid crystals [22] and polyaromatic compounds [23]. We chose a mechanical vibrating mortar grinder as a close simulation of manual grinding by mortar and pestle but in a much more controllable and reproducible manner. The fluorination of 2-naphthol with two molar equivalents of F-TEDA-BF_4_ on a 1 mmol scale after 2 h of grinding resulted in the formation of a mixture of two products, 1-fluoro-2-naphthol **3a** and 1,1-difluoronaphthalen-2(1*H*)-one **4a** with 9% and 4% yields, respectively (Table 1, Scheme 2). The ratio of difluoroketone to monofluoronaphthol, **4a**:**3a**, was 2.25 in this case, with 13% overall conversion of the aromatic substrate. The **4a**:**3a** ratio indicates the efficiency of the fluorinating reagent consumption. Full conversion of the starting material was achieved only after 4 days of grinding.

To improve the rate of fluorination, we decided to try a liquid-assisted grinding (LAG) approach with the addition of small amounts of solvents as grinding additives. As we demonstrated previously, the products of fluorination of 2-naphthols can be easily separated and purified by vacuum sublimation [14]. Therefore, non-volatile solvents were our choice as grinding additives. Ionic liquids represent a class of non-volatile [24], recyclable solvents that attract much attention as solvents, co-solvents, and catalysts [25,26,27,28].

In the second step, we chose PEG-400 as a representative non-volatile molecular grinding additive that has already been employed for green fluorination [29,30]. The addition of 10 mol% of relatively non-polar PEG-400 led to a moderate increase of the conversion of starting 2-naphthol to 22%. At the same time, 1-fluoro-2-naphthol became the major product, and the **4a**:**3a** ratio was 0.17. One of the possible explanations for this is the prevention of the dissociation of 2-naphthol and 1-fluoro-2-naphthol that led to lower activity and better selectivity of monofluorination (Table 1).

The addition of 10 mol% of low volatile but polar DMF to the mixture of **1** and **2a** led to a slightly higher conversion of 2-naphthol. The **4a**:**3a** ratio obtained demonstrates the prevalence of naphthalenone **4a** as the main product (Table 1, entry 3).

To further investigate the effects of additives on the reaction outcome, we used several ionic liquids, varying both cations and anions. First of all, we studied 1-butyl-3-methylimidazolium-based ILs containing the complex fluoride anions BF_4_ and PF_6_ (Table 1, entries 4 and 5). In both cases, the conversion of 2-naphthol was only slightly higher than in the absence of additive, and 1-fluoro-2-naphthol **3a** was obtained as the major product. Additionally, the selectivity of monofluorination was much lower than in the case of PEG-400 additive. In contrast, the triflate (OTf) IL demonstrated an acceleration of fluorination with the formation of ketone **4a** as the major product (Table 1, entry 6). Finally, we investigated 1-butyl-3-methylimidazolium bis(trifluorometanesulfonimide) (NTf_2_) as a hydrophobic ionic liquid additive. The conversion of 2-naphthol was higher than in cases of the complex anions but slightly lower than in the presence of the triflate IL (Table 1, entry 7).

To elucidate the effect of cation in IL, we repeated the fluorination with the addition of 1-ethyl-3-methylimidazolium ionic liquids. An increased conversion was achieved in the case of the triflate IL (Table 1, entry 9). Then, we investigated the influence of fluorosulfonimide (FSI) and trifluoromethylsulfonimide (NTf_2_) anions. A relatively high selectivity of difluorination was achieved, comparable with the fluorination without additives (emim NTf_2_), or even higher (emim FSI).

To further study the effect of cation in IL, we carried out fluorination in the presence of 1-butyl-2,3-dimethylimidazolium, 1-butyl-1-methylpyrrolidinium, 1-butylpyridinium bis(trifluoromethanesulfonimides), and 1-butyl-1-methylpyrrolidinium triflate. In all cases, the obtained conversions of 2-naphthol were lower than for bmim- or emim-based ILs (Table 1, entries 12–15).

**Figure 1:**
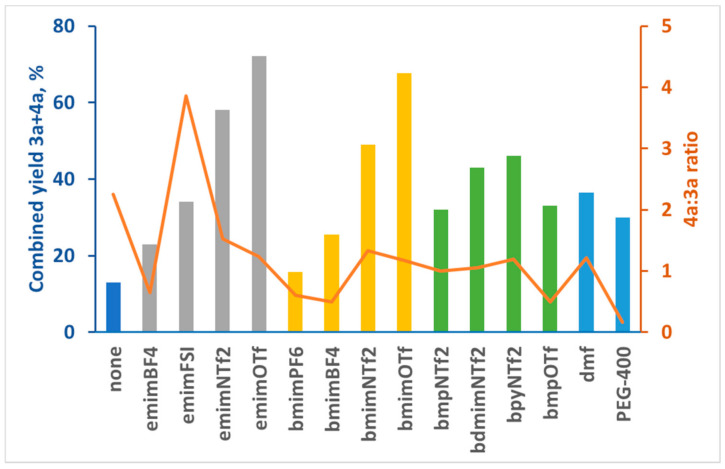
Effect of grinding additives on the electrophilic fluorination of 2-naphthol.

The performed screening revealed that emim and bmim ILs with fluorous anions OTf and NTf_2_ work best as grinding additives. The obtained results are shown on Figure 1.

To elucidate the effect of additive quantity, we chose two emim-based ILs with OTf and NTf_2_ anions that most significantly increase the rate of fluorination (Table 2). The increase of loadings of IL additive to 15% led to an expected growth of conversion of 2-naphthol. But for triflate ILs, such an increase of conversion was not accompanied with improved selectivity; for the run with 15 mol% of emim OTf, **4a** and **3a** formed in almost equal quantities. On the contrary, an increase of emim NTf_2_ loading led to a pronounced increase of not only the conversion of 2-naphthol, but also the **4a**:**3a** ratio, which indicated a higher conversion of fluorinating reagent. Therefore, we chose emim NTf_2_ for further reaction optimization. Moreover, this ionic liquid is particularly hydrophobic; hence, it should prevent or at least diminish the absorption of ambient moisture during synthesis.

To define an optimal time for the synthesis of the difluorinated product, we monitored the fluorination of 2-naphthol in the presence of 10 mol% of emim NTf_2_ until the fluorinating reagent was completely consumed (Table 2). Most of the aromatic substrate (>80%) was consumed after 4 h, with the formation of a mixture of mono- and difluorinated products. High selectivity towards difluoroketone **4a** was achieved after 20 h, and after 24 h, only trace amounts of **3a** could be detected spectroscopically.

We recently reported the dry method for the synthesis of organofluorine compounds based on mechanochemical fluorination followed by vacuum sublimation of the product directly from the reaction mixture.

A combination of ionic liquid-assisted grinding with vacuum sublimation of products resulted in improved preparative yields of 1,1-difluoronaphthalen-2(1*H*)-ones in comparison with previously reported manual grinding procedures. We consider Sheldon’s E-factor to analyze the outcome of the improved mechanochemical procedure. The E(nvironmental)-factor is one of the green chemistry metrics, calculated as a ratio of mass of waste/mass of product expressed as kgs/kg [31]. On a 1 mmol scale, we isolated unsubstituted 1,1-difluoronaphthalen-2(1*H*)-one with 88% yield, and the E-factor was determined to be as low as 4.6. The preparative yield of 6-bromo-1,1-difluoronaphthalen-2(1*H*)-one on the same molar loadings was even higher—94%, with an even lower E-factor of 3.0.

To prove the scalability of the reaction studied, we carried out the fluorination of 2-naphthol **2a** on a 10 mmol scale. Fluorination of 1.44 g of 2-naphthol with 2-fold excess of F-TEDA-BF_4_ in the presence of emim NTf_2_ followed by vacuum sublimation of the product resulted in 68% preparative yield of the target 1,1-difluoronaphthalen-2(1*H*)-one **4a** with 99% purity by GC. Even with a large excess of fluorinating reagent, the E-factor for this process could be estimated as 6.7.

**Scheme 3:**
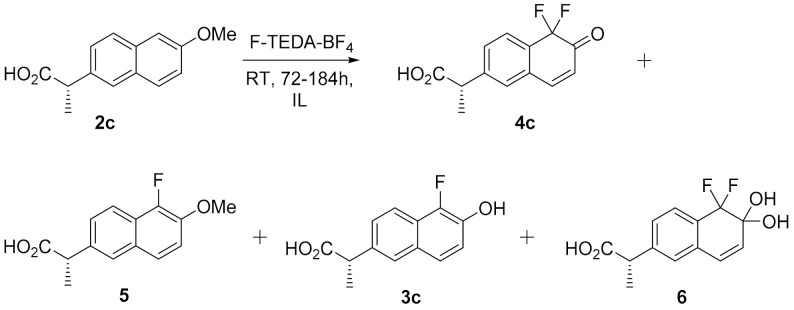
Mechanochemical electrophilic fluorination of naproxen in the presence of emim NTf_2_.

Further, we decided to adapt the fluorination procedure to the late-stage functionalization of a practically important molecule. Thus, we performed a modification of naproxen, a widely used non-steroidal anti-inflammatory drug (NSAID) that contains a fragment of 2-naphthol ether. Phenol ethers are typically less reactive in electrophilic aromatic substitution reactions. Thus, the fluorination of naproxen in the presence of emimNTf_2_ required 72 h of grinding to complete. The fluorination of naproxen led to the formation of a complex mixture of monofluoronaproxen **5**, demethylated monofluoro derivative **3c**, the target ketone **4c,** and its hydrated form **6** (Scheme 3). After the completion of the reaction, the reaction mixture consisted of naphthalenone **4c** and dihydroxy derivative **6**. Drying of the ethereal solution resulted in obtaining pure **4c** with 70% yield. The use of more hygroscopic emim OTf led to the isolation of a 7:1 mixture of **4c** and **6** with 72% overall yield.

Finally, we investigated the ionic liquid-assisted fluorination of 1,3-dicarbonyl compounds. The fluorination of 2-methylcyclopenta-1,3-dione **7** with an equimolar amount of F-TEDA-BF_4_ on a 1 mmol scale in the presence of emim NTf_2_ led to complete conversion of the starting material to the expected 2-fluoro-2-methylcyclopenta-1,3-dione **8**. Vacuum sublimation from the reaction mixture yielded pure **8** with 75% isolated yield; and the overall process has an E-factor of 4.2.

**Scheme 4:**
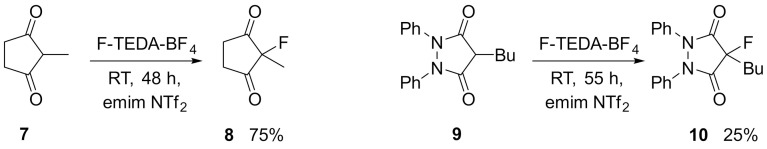
Mechanochemical electrophilic fluorination of 2-methylcyclopenta-1,3-dione and phenylbutazone in the presence of emim NTf_2_.

This procedure was employed for the fluorination of another NSAID, phenylbutazone **9** (Scheme 4). Already after 24 h of grinding, a 23% conversion to the corresponding product **10** was observed. Unfortunately, further grinding resulted in the formation of a viscous mixture that prevented efficient mixing of the reagents. After 55 h, only 25% of the target fluoro derivative **10** was formed.

## 3. Discussion

**Figure 2:**
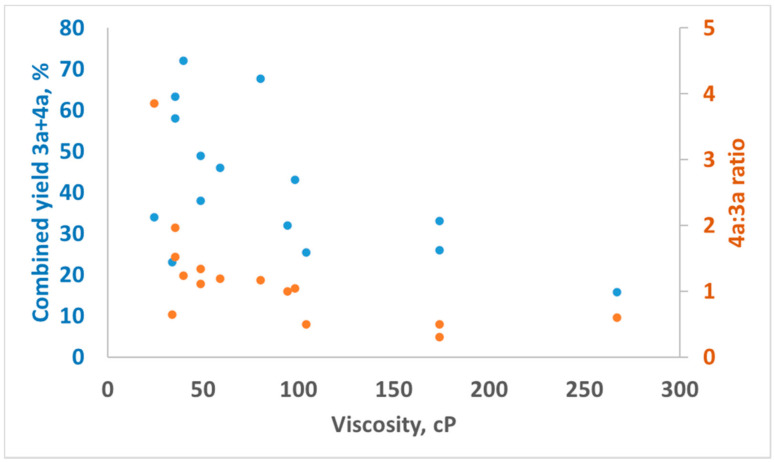
Effect of viscosity of grinding additives on the electrophilic fluorination of 2-naphthol.

Solvent-free synthesis typically suffers from the insufficient diffusion rate of the reagents toward each other. During high-speed mixing, a local increase of temperature at the impact sites can result in local melting that speeds up the reaction. But such an intense impact can also cause the degradation of organic compounds. The use of milder grinding techniques results in low reaction rates. The addition of small amounts of liquid additives typically speeds up mechanochemical transformations. The possible mechanism of such an effect is the formation of a thin interface layer on the grain boundaries of solid reagents or solutions of both reagents at the interface. In that case, a lower viscosity of an additive should result in a higher reaction rate. As demonstrated in Figure 2, while no distinct correlation has been obtained, the lower viscosities of ionic liquids are typically associated with higher conversions of both aromatic substrate and fluorinating reagent.

## 4. Materials and Methods

^1^H-, ^13^C- and ^19^F-NMR spectra were recorded in CDCl_3_ or acetone-*d*_6_ on a Bruker AV-300 spectrometer (Karlsruhe, Germany) and chemical shifts are given in ppm relative to TMS and CFCl_3,_ respectively, with C_6_F_6_ (^19^F, −162.9 ppm) or residual solvent signals (^1^H, ^13^C) as secondary external standards. GC/MS spectra were recorded on Agilent 6890 instrument operating at 70 eV with MSD Agilent 5973 (Santa Clara, CA, USA). High resolution mass spectra (HRMS) were measured using Thermo Fisher Scientific Double Focusing System (DFS) Magnetic Sector high resolution mass-spectrometer (Santa Clara, CA, USA) operating at 70 eV electron ionization and 200 °C ion source temperature. All reactants were obtained from commercial sources and used without further purification (F-TEDA-BF_4_ > 96%, 2-naphthol > 95%, 6-bromo-2-naphthol > 98%, ionic liquids > 99%, 2-Methyl-1,3-cyclopentanedione > 99%, *(S)*-naproxene, and phenylbutazone, pharm. grade). The spectral data of the products obtained were consistent with literature data. Mechanochemical experiments were carried out in the vibratory mortar grinder MLW KM1 (Leipzig, Germany) equipped with an agate grinding ball (198 g, Ø52 mm (See Supplementary Materials, Figure S1)). The experiments were carried out at 20–21 °C and 50–55% relative humidity (RH).

### 4.1. General Procedure for Screening of Additives in Liquid-Assisted Grinding

A mixture of 2-naphthol (144 mg, 1 mmol), F-TEDA-BF_4_ (709 mg, 2 mmol), and the additive (0.1–0.15 mmol, see Table 1 and Table 2) was manually homogenized for 1 min in a porcelain mortar. The reaction mixture was then transferred to the vibratory mortar grinder and was ground for the time required (Table 1 and Table 2). After the end of the process, the resulting mixture was transferred to a Soxhlet apparatus and continuously extracted with ether. The solvent was removed in vacuo to yield crude products which were analyzed by ^1^H-, ^19^F-NMR spectroscopy and GC/MS. Alternatively, the reaction mixture was transferred to a 1.5 mL centrifuge tube and extracted three times with CDCl_3_ by intensive vortex mixing followed by centrifugating. The combined extracts containing the aromatic substrate, the products, and the grinding additive were directly analyzed by ^1^H-, ^19^F-NMR spectroscopy. Yields were determined by comparing the intensities of signals of products in NMR spectra relative to the signal intensity of the additive or the weighted standard added (emim NTf_2_).

### 4.2. General Procedure for Fluorination Followed by Vacuum Sublimation 

2-Naphthol (144 mg, 1 mmol), F-TEDA-BF_4_ (709 mg, 2 mmol), and emim NTf_2_ (39 mg, 0.1 mmol, 10 mol%) were homogenized manually by mortar and pestle for 1 min and transferred to the vibratory mortar grinder. The mixture was ground at room temperature until the fluorinating reagent was fully consumed (10 days). Then, the reaction mixture was transferred to a sublimation apparatus and the target 1,1-difluoronaphthalen-2(1*H*)-one was sublimed in vacuo at 70 °C; 1,1-Difluoronaphthalen-2(1*H*)-one (**4a**); yellow needles (158 mg, 88%); m.p. 50–51 °C; E-factor 4.6.

6-Bromo-1,1-difluoronaphthalen-2(1*H*)-one (**4b**) was obtained as yellow needles (244 mg, 94%); m.p. 67–68 °C [14]; E-factor 3.0.

2-Fluoro-2-methylcyclopenta-1,3-dione (**8**); colorless crystals (98 mg, 75%); E-factor 4.2.

### 4.3. General Procedure for the Scale-Up of Fluorination of 2-Naphthol with F-TEDA-BF_4_ Followed by Vacuum Sublimation 

2-Naphthol (1.44 g, 10 mmol), F-TEDA-BF_4_ (7.78 g, 22 mmol), and emim NTf_2_ (196 mg, 0.5 mmol, 5 mol%) were homogenized manually by mortar and pestle for 1 min and then transferred to the vibratory mortar grinder. The mixture was ground at room temperature until the fluorinating reagent was fully consumed (36 h) as determined by ^1^H- and ^19^F-NMR. Then, the reaction mixture (9 g) was transferred to a sublimation apparatus and the target 1,1-difluoronaphthalen-2(1*H*)-one was sublimed in vacuo at 70 °C; 1,1-Difluoronaphthalen-2(1*H*)-one (**4a**); yellow needles (1.23 g, 68%); m.p. 50–51 °C; E-factor 6.7.

### 4.4. Fluorination of Naproxen

(*2S*)-2-(6-methoxy(2-naphthyl))propanoic acid (naproxen) (115.2 mg, 0.5 mmol), F-TEDA-BF_4_ (390.0 mg, 1.1 mmol), and emim NTf_2_ (19.8 mg, 0.05 mmol) were ground in the vibratory mortar grinder for 72 h with occasional manual mixing of the reaction mixture. After the end of the grinding time, the reaction mixture was extracted with ether, and the extract was washed with water to remove the IL additive, dried, and evaporated in vacuo; 2-(5,5-difluoro-6-oxo-5,6-dihydronaphthalen-2-yl)propionic acid was obtained (88 mg, 70%) [32].

## 5. Conclusions

An efficient and ecologically benign method for fluorination of activated aromatic compounds and 1,3-dicarbonyl compounds was developed using small amounts of ionic liquids as grinding additives. The influence of cation and anion of ionic liquids on the rate and selectivity of solvent-free fluorination of 2-naphthol was investigated. Emim-derived ionic liquids with the fluorous anions OTf or NTf_2_ were shown to be the most efficient promoters. A solvent-free fluorination of 2-naphthol and 6-bromo-2-naphthol was performed, followed by vacuum sublimation for the isolation of fluorinated products, with high yields and purity. The fluorination procedure was adapted for the fluorination of 1,3-dicarbonyl compounds. The low E-factor values of 3–6.7 were achieved for the proposed dry fluorination method. Fluorination of the practically useful non-steroidal anti-inflammatory drugs naproxen and phenylbutazone was also performed.

## Figures and Tables

**Table 1:** The influence of grinding additives on the rate and selectivity of fluorination of 2-naphthol with F-TEDA-BF_4_.

Entry	Additive ^1^	Products Yields ^2^	Conversion of 2a, %	4a:3a
1	None	**4a** (9%), **3a** (4%)	13	2.3
2	PEG-400	**4a** (4%), **3a** (26%)	30	0.2
3	DMF	**4a** (20%), **3a** (16%)	36	1.2
4	bmim BF_4_	**4a** (8%), **3a** (17%)	25	0.5
5	bmim PF_6_	**4a** (6%), **3a** (10%)	16	0.6
6	bmim OTf	**4a** (36%), **3a** (31%)	67	1.2
7	bmim NTf_2_	**4a** (28%), **3a** (21%)	49	1.3
8	emim BF_4_	**4a** (9%), **3a** (14%)	23	0.6
9	emim OTf	**4a** (40%), **3a** (32%)	72	1.2
10	emim NTf_2_	**4a** (35%), **3a** (23%)	58	1.5
11	emim FSI	**4a** (27%), **3a** (7%)	34	3.9
12	bmp OTf	**4a** (11%), **3a** (22%)	33	0.5
13	bmp NTf_2_	**4a** (16%), **3a** (16%)	32	1.0
14	bdmim NTf_2_	**4a** (22%), **3a** (21%)	43	1.0
15	bpy NTf_2_	**4a** (25%), **3a** (21%)	46	1.2

^1^ 1 mmol of **2a**, 2 mmol of **1**, 0.1 mmol of additives, 2 h, RT. ^2^ Yields are determined by ^19^F-NMR using internal standard.

**Table 2:** The influence of grinding additives on the rate and selectivity of fluorination of 2-naphthol with F-TEDA-BF_4_.

Entry	Additive	IL Amount, mol%	Reaction Time, h	Products Yields ^1^	4a:3a
1	emim OTf	10	2	**4a** (40%), **3a** (32%)	1.2
2	emim NTf_2_	10	2	**4a** (35%), **3a** (23%)	1.5
3	emim OTf	15	2	**4a** (40%), **3a** (37%)	1.1
4	emim NTf_2_	15	2	**4a** (69%), **3a** (16%)	4.1
5	emim NTf_2_	17	2	**4a** (77%), **3a** (5%)	14.0
6	emim OTf	10	4	**4a** (62%), **3a** (21%)	2.9
7	emim NTf_2_	10	4	**4a** (59%), **3a** (21%)	2.8
8	emim NTf_2_	10	20	**4a** (89%), **3a** (2%)	44.5
9	emim NTf_2_	10	24	**4a** (87%), **3a** (1%)	87

^1^ Yields are determined by ^19^F-NMR using internal standard.

## Data Availability

The data presented in this study are available in the present article.

## Supplementary Materials

The following are available online, Figure S1: mechanochemical setup.

## Sample Availability

Samples of the compounds **4a**–**c** are available from the authors.
