# Supplementary material for: Ionic Liquid-Assisted Grinding: An Electrophilic Fluorination Benchmark
Source: Molecules. 2021 Sep 23;26(19):5756. doi: 10.3390/molecules26195756 (PMC8510021; doi:10.3390/molecules26195756)
Supplement: Supplementary file 1 [file molecules-26-05756-s001.zip › molecules-1386105-supplementary.pdf]

Supplementary Information

Ionic liquids-assisted grinding: an electrophilic fluorination benchmark

Pavel A. Zaikin[1,*], Ok Ton Dyan[1,2], Innokenty R. Elanov[1] and Gennady I. Borodkin[1,2]

Experimental Section

^{1}H, ^{13}C and ^{19}F NMR spectra were recorded in $CDCl_3$ or acetone-*d*$_6$ on Bruker AV-300 spectrometers and chemical shifts are given in ppm relative to TMS and $CFCl_3$ respectively with C_6F_6 (^{19}F, -162.9 ppm) or residual solvent signals (^{1}H, ^{13}C) as secondary external standards. GC/MS spectra were recorded on an Agilent instrument operating at 70eV. High resolution mass spectra (HRMS) were measured using DFS instrument. IR spectra were recorded on Bruker Vector 22 spectrometer and UV-Vis spectra were recorded on Varian Cary 5000 spectrophotometer (lgε is indicated in brackets). All reactants were obtained from commercial sources and used without further purification. Spectral data of products obtained were consistent with literature data. Mechanochemical experiments were carried out in a vibratory mortar grinder MLW KM1 equipped with an agate grinding ball 198 g, Ø52 mm. Experiments were carried out at 20-21°C and 50-55% Relative humidity (RH)

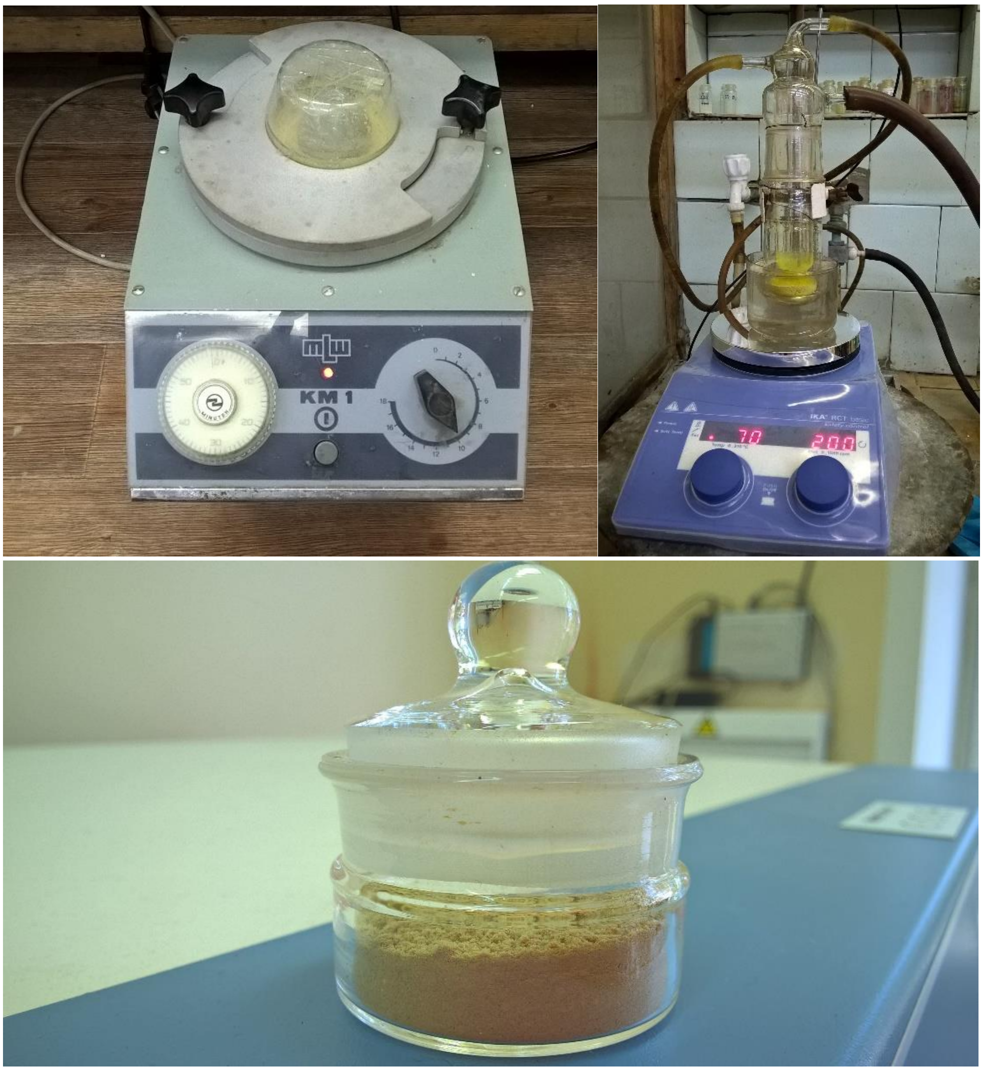

Figure S1. A. Vibratory mortar grinder MLW KM1 with grinding ball and reaction mixture. B. Sublimation setup. C. Final product
